# Association of Red Meat and Poultry Consumption With the Risk of Metabolic Syndrome: A Meta-Analysis of Prospective Cohort Studies

**DOI:** 10.3389/fnut.2021.691848

**Published:** 2021-07-08

**Authors:** Hongbin Guo, Jun Ding, Jieyu Liang, Yi Zhang

**Affiliations:** ^1^Department of Orthopedics, Xiangya Hospital, Central South University, Changsha, China; ^2^Changsha Social Work College, Changsha, China

**Keywords:** red meat, poultry, metabolic syndrome, meta-analysis, prospective cohort study

## Abstract

**Objective:** This study aims to investigate the association of red meat (processed and unprocessed) and poultry consumption with the risk of metabolic syndrome (MetS).

**Methods:** Prospective cohort studies on the association of red meat (processed and unprocessed) and poultry consumption with the risk of MetS were identified by comprehensive literature search in the PubMed, Web of Science, and Embase databases up to March 2021. The pooled relative risk (RR) of MetS with 95% CIs for the highest vs. lowest category of red meat or poultry consumption was extracted for meta-analysis.

**Results:** A total of nine prospective cohort studies were included in this study. Among them, eight studies were identified for red meat consumption. The overall multi-variable adjusted RR demonstrated that red meat consumption was associated with a higher risk of MetS (RR = 1.35, 95% CI: 1.13–1.62; *P* = 0.001). Moreover, four and three studies were specifically related to processed and unprocessed red meat consumption, respectively. Both processed (RR = 1.48, 95% CI: 1.11–1.97; *P* = 0.007) and unprocessed red meat (RR = 1.32, 95% CI: 1.14–1.54; *P* = 0.0003) consumption was associated with a higher risk of MetS. With regard to poultry consumption, three studies were included. The overall multi-variable adjusted RR suggested that poultry consumption was associated with lower risk of MetS (RR = 0.85, 95% CI: 0.75–0.97; *P* = 0.02).

**Conclusions:** The current evidence indicates that red meat (processed and unprocessed) consumption is associated with a higher risk of MetS, whereas, poultry consumption is associated with a lower risk of MetS. More well-designed randomized controlled trials are still needed to address the issues further.

## Introduction

Metabolic syndrome (MetS) is defined as the clustering of at least three of the five following metabolic alterations: high serum triglyceride, low high-density lipoprotein cholesterol, increased fasting plasma glucose, elevated waist circumference, and elevated blood pressure (1). In the developed world, around 25% of the population is suffering from MetS, and the prevalence of this condition is still increasing exponentially (2). MetS is considered as an important public health issue in the 21st century. It is well-known that many etiologic factors are associated with MetS [obesity (3), alcohol drinking (4), and cigarette smoking (5)]. Among them, diet is considered as an important factor (6, 7).

As an important part of the global dietary structure, meat is rich in protein, fat, iron, zinc, and vitamin B_12_ (8, 9). According to Healthy Eating Index-2015 (HEI-2015), a lean part of red meat and poultry was included in protein foods, and a healthy eating pattern with a variety of protein foods (such as red meat and poultry) was also recommended by the latest US Dietary Guidelines recommendations (10). However, some other parts of red meat and poultry (e.g., fats) were classified as non-protein, which could not be ignored for their health issues. Indeed, meat consumption was reported to be associated with digestive system disease (11), cardiovascular disease (12), type 2 diabetes (13), and cancer (14) by several observational studies. Thus, it is important and interesting to further investigate the relationship between meat consumption and MetS.

Generally speaking, “red” meat refers to beef, pork, horse, veal, deer, and lamb, whereas, poultry is considered as “white” meat. “Processed red meat” mainly indicates red meat products with ingredients (sausages, cold cuts, and others). Of note, salt is always added to extend its shelf life (15). Therefore, the effect of meat on MetS may vary greatly. In 2018, the meta-analysis of an observational study has examined the issues above (16). However, it combined the results from observational studies together, which may raise unpredictable heterogeneity. Moreover, it failed to demonstrate any association between red meat consumption and the risk of MetS (only three prospective cohort studies were included), and unprocessed red meat consumption was not considered either. Therefore, a meta-analysis of prospective cohort studies with detailed meat specification is important and necessary to be performed. As far as we know, a number of prospective cohort studies have examined the association of red meat and poultry consumption with the risk of MetS (17–25). However, no final conclusion can be drawn. Taken together, this meta-analysis was, therefore, employed to further investigate the issues above.

## Methods

### Search Strategy

This meta-analysis was conducted in accordance with the Preferred Reporting Items for Systematic Reviews and Meta-analyses (PRISMA) guidelines (26). We searched the PubMed, Web of Science, and Embase databases in March 2021 by a series of keywords related to metabolic syndrome (“metabolic syndrome”), red meat (“meat”), poultry (“poultry”) and prospective cohort (“cohort,” “follow-up,” “incidence,” “incident,” “prospective,” “prognosis,” “prognostic” and predict”). No restrictions for language were set in the search strategy. Titles and abstracts were screened to identify eligible studies. Then, full articles were also read to include eligible studies.

### Study Selection

The titles, abstracts, and full texts of all potential studies were reviewed by two researchers (YZ and JD) independently. Criteria for a study to be included were listed as follow: (1) prospective cohort studies; (2) the exposure of interest was red meat and poultry consumption; (3) the study outcome included the risk of MetS; and (4) hazard ratio (HR) or RR with 95% CI reported. Exclusion criteria were listed as follows: (1) duplicated studies; (2) irrelevant studies; (3) reviews, letters, or case reports; (4) randomized controlled trials; and (5) non-human studies.

### Data Extraction

Two independent reviewers (YZ and JD) extracted the data. The following information was collected: first author, year of publication, location, age, gender, sample size, follow-up, adjustments, exposure assessment, category of exposure, effect estimates, and diagnostic criteria of MetS. The effect estimates adjusted for the maximum number of confounding variables with 95% CIs for the highest vs. lowest level were extracted. Red meat refers to the combination of processed and unprocessed red meat. The majority of included studies reported estimates for red meat (processed or unprocessed) or poultry (white meat) directly. However, Asghari et al. reported the estimated effect as hamburger, sausages, and beef, which were, therefore, combined as processed red meat (20). Moreover, Shang translated egg consumption into protein intake, and the estimate effect was utilized directly (21).

### Statistical Analysis

The RR of the association of red meat and poultry consumption with MetS was considered as the outcome. The percentage of the total variation across studies due to heterogeneity was examined by the I^2^ statistic (I^2^ > 50% was considered heterogeneity). A random effects model was utilized when significant heterogeneity was obtained; otherwise, a fixed effects model was employed. Publication bias was evaluated by both Begg's and Egger's tests (27). STATA version 11.0 (StataCorp LP, College Station, Texas) was performed in all statistical analyses. A *p*-value < 0.05 was accepted as statistically significant. Moreover, we conducted a subgroup analysis for follow-up, diagnostic criteria of MetS, geographical region, sample size, adjustment of BMI, energy intake, and physical activity.

## Results

### Study Identification and Selection

**Figure 1:**
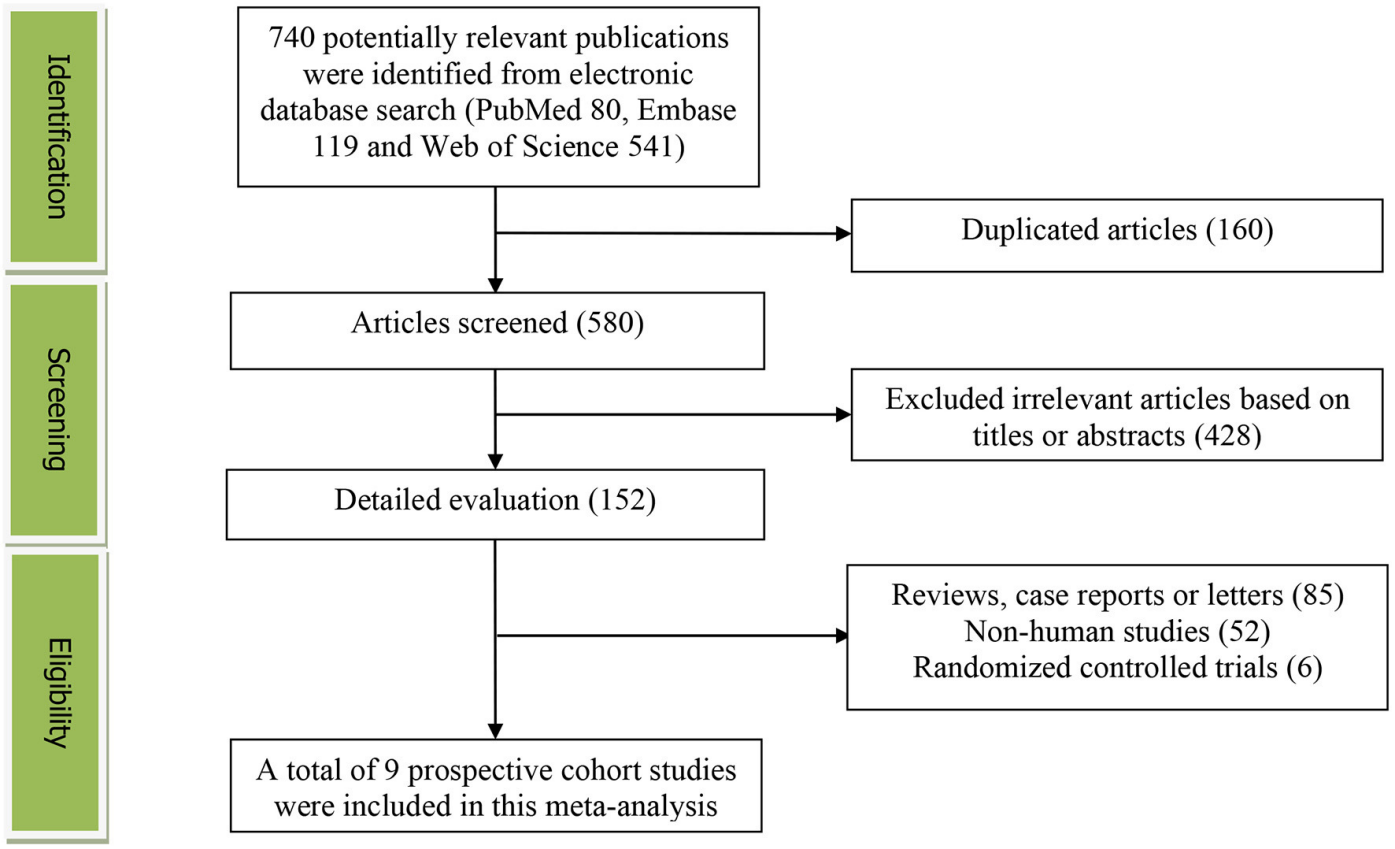
The detailed flow diagram of the study identification and selection in this meta-analysis.

The detailed flow diagram of study identification and selection is presented in Figure 1. Initially, a total of 740 potentially relevant articles (PubMed 80, Embase 119, and Web of Science 541) were retrieved, and 580 articles were screened by titles and abstracts after eliminating 160 duplicated articles. We first excluded 428 irrelevant studies. Thereafter, 85 reviews, case reports or letters, six randomized controlled trials and 52 non-human studies were removed. Finally, we identified nine prospective cohort studies for this meta-analysis.

### Study Characteristics

The characteristics of the included studies are shown in Table 1. These studies, which involved a total of 21,869 participants, were published between 2006 and 2021. Five studies were performed in Asian countries [Korea (19), Iran (20, 23, 25), and China (24)] and two were from Spain (18, 22). The other two studies were from Brazil (17) and Australia (21). Both male and female participants were considered in all the studies. Red meat and poultry consumption was assessed by a food frequency questionnaire (FFQ) in all studies. With regard to the diagnostic criteria of MetS, National Cholesterol Education Program-Adult Treatment Panel (NCEP-ATP III) was utilized in four studies (17, 18, 21, 23), and a joint interim statement (JIS) was used in three studies (19, 22, 24), respectively. Moreover, two studies employed Cook's criteria in children and adolescents (20, 25).

**Table 1:** Characteristics of prospective cohort studies included in this meta-analysis.

**First author year of publication**	**Location**	**Age years**	**Gender (%)**	**Sample size**	**Follow-up (year)**	**Adjustments**	**Exposure assessment**	**Category of Exposure**	**Effect estimates for MetS (95% CI)**	**Diagnostic criteria of MetS**
Damião 2006 (17)	Brazil	40–79	Both	151	7	Age, sex, physical activity, smoking, education level, alcohol, total energy intake, total fat intake, and fried foods	FFQ	Red meat		NCEP-ATP III
								Tertile 1	1	
								Tertile 2	1.84 (0.51, 6.67)	
								Tertile 3	3.18 (0.87, 11.5)	
								Poultry		
								Tertile 1	1	
								Tertile 2	2.57 (0.75, 8.83)	
								Tertile 3	1.36 (0.38, 4.78)	
Babio 2012 (18)	Spain	55–80	Both	870	1	Age, sex, smoking, BMI, physical activity, total energy intake, dietary alcohol, fiber, magnesium, and potassium	FFQ	Red meat		NCEP-ATP III
								Quartile 1	1	
								Quartile 2	1.10 (0.50, 2.70)	
								Quartile 3	2.70 (1.30, 7.20)	
								Quartile 4	2.70 (1.10, 6.80)	
								Unprocessed red meat		
								Quartile 1	1	
								Quartile 2	NA	
								Quartile 3	NA	
								Quartile 4	2.20 (1.00, 5.10)	
								Processed red meat		
								Quartile 1	1	
								Quartile 2	NA	
								Quartile 3	NA	
								Quartile 4	2.50 (1.00, 6.20)	
Baik 2013 (19)	Korea	40–69	Both	5251	6	Age, sex, income, occupation, education, smoking status, alcohol intake, quartiles of MET-hours/day, study sites, FTO genotypes, quartiles of energy intake, and quintiles of food groups or food items.	FFQ	Red meat		JIS
								Quintile 1	1	
								Quintile 2	1.05 (0.88, 1.26)	
								Quintile 3	1.17 (0.95, 1.45)	
								Quintile 4	0.96 (0.75, 1.24)	
								Quintile 5	1.01 (0.79, 1.29)	
								Poultry		
								Quintile 1	1	
								Quintile 2	NA	
								Quintile 3	NA	
								Quintile 4	1.08 (0.93, 1.25)	
								Quintile 5	0.88 (0.71, 1.09)	
Asghari 2015 (20)	Iran	6–18	Both	424	3.6	Age, sex, total energy intake, physical activity, dietary fiber, family history of diabetes, and meat, poultry, fish, grains,legumes, and BMI	FFQ	Processed red meat		Cook criteria
								Quartile 1	1	
								Quartile 2	1.06 (0.53, 2.13)	
								Quartile 3	1.48 (0.87, 2.51)	
								Quartile 4	2.38 (1.40, 4.05)	
Shang 2016 (21)	Australia	49.2	Both	5324	11.2	Age, gender, follow-up period, ethnicity, socio-economic status, physical activity, smoking, alcohol intake, BMI, WC, BP, plasma TC, glucose at baseline, glycaemic index,	FFQ	Red meat		NCEP-ATP III
								Quartile 1	1	
								Quartile 2	1.17 (0.85, 1.61)	
								Quartile 3	1.27 (0.91, 1.78)	
								Quartile 4	1.47 (1.01, 2.15)	
						consumption of energy, fiber, sodium, potassium, magnesium, vitamin C, vitamin E, saturated fat, monounsaturated fat, polyunsaturated fat, and trans fat				
Becerra-Tomás 2016 (22)	Spain	55–80	Both	1868	3.2	Sex, age, leisure time physical activity, BMI, current smoker, former smoker, vegetables, fruit, legumes, cereals, fish, dairy products, alcohol, biscuits, olive oil, nuts, abdominal obesity, hypertriglyceridemia, low HDL-cholesterol, hypertension, and high fasting plasma glucose.	FFQ	Red meat		JIS
								Tertile 1	1	
								Tertile 2	0.98 (0.82, 1.17)	
								Tertile 3	1.46 (1.22, 1.74)	
								Unprocessed red meat		
								Tertile 1	1	
								Tertile 2	0.86 (0.72, 1.02)	
								Tertile 3	1.27 (1.06, 1.52)	
								Processed red meat		
								Tertile 1	1	
								Tertile 2	1.06 (0.89, 1.26)	
								Tertile 3	1.37 (1.15, 1.62)	
								Poultry		
								Tertile 1	1	
								Tertile 2	0.74 (0.63, 0.88)	
								Tertile 3	0.83 (0.70, 0.99)	
Esfandiar 2019 (23)	Iran	>18	Both	4653	3.8	Age, sex, baseline BMI, educational level, smoking status, total energy intake, fiber, saturated fat, sodium, vitamin C, and magnesium intakes	FFQ	Red meat		NCEP-ATP III
								Quartile 1	1	
								Quartile 2	0.86 (0.55, 1.26)	
								Quartile 3	0.96 (0.68, 1.28)	
								Quartile 4	0.87 (0.56, 1.24)	
Huang 2020 (24)	China	18–75	Both	2797	6	Age, gender, regions and household income level, BMI, urbanicity index, smoking, drinking alcohol, physical activity, and TEI, dietary fiber, fat, carbohydrate, usual intake of vegetables and fruits	FFQ	Red meat		JIS
								Quartile 1	1	
								Quartile 2	1.03 (0.79, 1.34)	
								Quartile 3	1.14 (0.87, 1.49)	
								Quartile 4	1.41 (1.05, 1.90)	
								Unprocessed red meat		
								Quartile 1	1	
								Quartile 2	1.03 (0.79, 1.34)	
								Quartile 3	1.24 (0.95, 1.63)	
								Quartile 4	1.37 (1.02, 1.85)	
								Processed red meat		
								Quartile 1	1	
								Quartile 2	1.14 (0.90, 1.45)	
								Quartile 3	1.13 (0.90, 1.42)	
								Quartile 4	NA	
Yuzbashian 2021 (25)	Iran	6–18	Both	531	6.6	Not mentioned	FFQ	Red meat		Cook criteria
								Non-red meat consumer	1	
								Replacement by red meat	1.55 (1.21, 1.97)	

### Red Meat Consumption and the Risk of MetS

**Figure 2:**
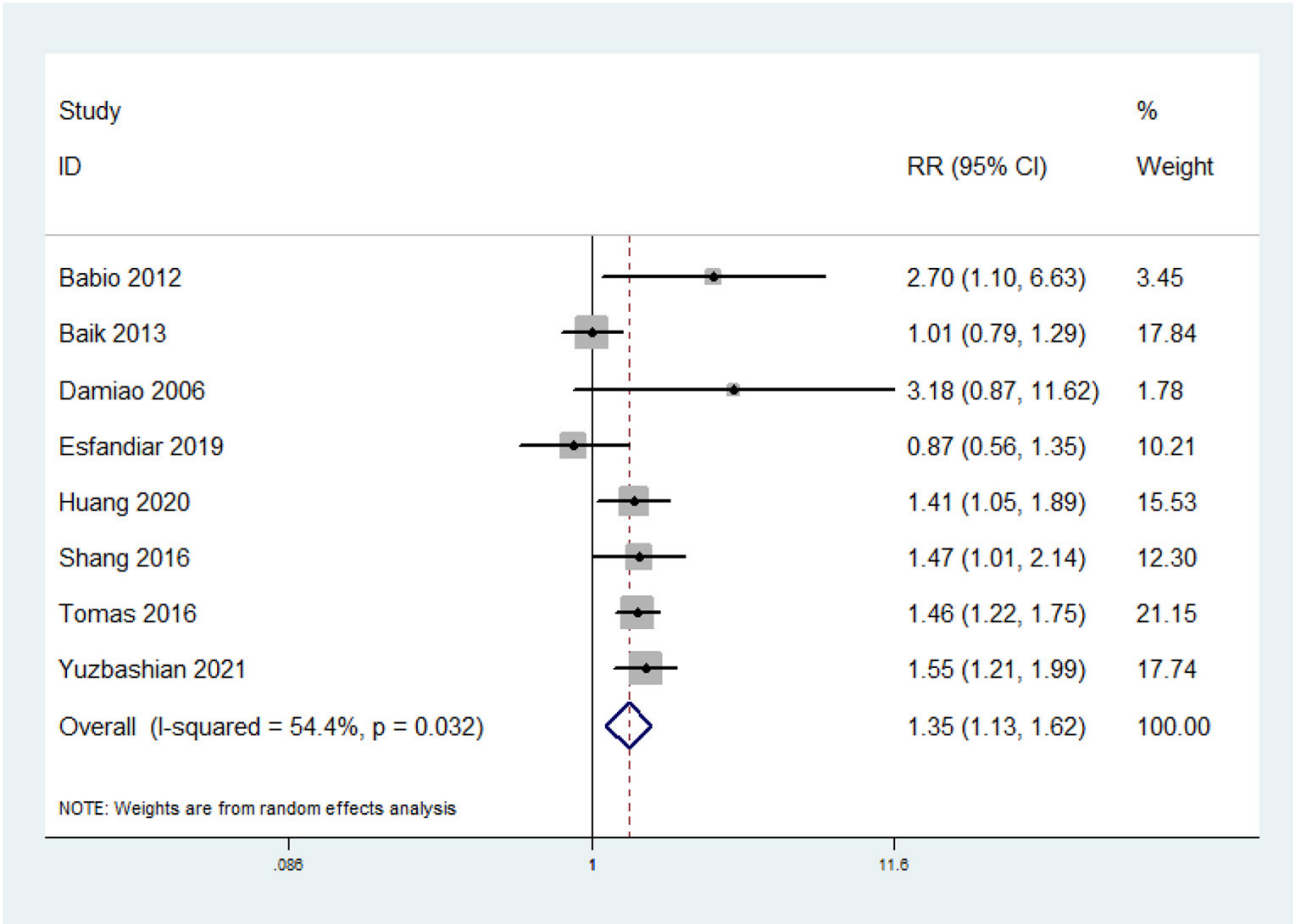
Forest plot of meta-analysis: overall multi-variable adjusted RR of MetS for the highest vs. lowest category of red meat consumption.

Eight prospective cohort studies were included. The overall multi-variable adjusted RR showed that red meat consumption was associated with higher risk of MetS (RR = 1.35, 95% CI: 1.13–1.62; *P* = 0.001) (Figure 2). We found a substantial level of heterogeneity among the studies (*P* = 0.032, I^2^ = 54.4%). No publication bias was observed according to the Begg's rank-correlation test (*P* = 0.386) and the Egger's test (*P* = 0.574). The results of subgroup analysis are presented in Table 2. The same results were obtained in > 5 years follow-up (RR = 1.36, 95% CI: 1.09–1.7; *P* = 0.006), non-NCEP ATP III (RR = 1.34, 95% CI: 1.12–1.62; *P* = 0.002), Non-Asia (RR = 1.51, 95% CI: 1.29–1.77; *P* < 0.001), adjustment of BMI (RR = 1.4, 95% CI: 1.23–1.6; *P* < 0.001), and physical activity studies (RR = 1.48, 95% CI: 1.29–1.71; *P* < 0.001).

**Table 2:** Subgroup analysis of relationship between red meat consumption and risk of MetS.

**Stratification**	**Number of studies**	**Pooled RR**	**95% CI**	***P*-value**	**Heterogeneity**
All	8	1.35	1.13, 1.62	*P* = 0.001	*P* = 0.03; I^2^ = 54%
**Follow-up**					
<5	3	1.36	0.85, 2.17	*P* = 0.20	*P* =0.03; I^2^ = 70%
>5	5	1.36	1.09, 1.70	*P* = 0.006	*P* = 0.08; I^2^ = 53%
**Diagnostic criteria of MetS**					
NCEP ATP III	4	1.51	0.91, 2.52	*P* = 0.11	*P* = 0.05; I^2^ = 62%
Non-NCEP ATP III	4	1.34	1.12, 1.62	*P* = 0.002	*P* = 0.06; I^2^ = 59%
**Geographical region**					
Asia	4	1.21	0.94, 1.56	*P* = 0.15	*P* = 0.03; I^2^ = 67%
Non-Asia	4	1.51	1.29, 1.77	*P* < 0.001	*P* = 0.39; I^2^ = 1%
**Sample size**					
<1,000	3	1.65	1.30, 2.08	*P* < 0.001	*P* = 0.30; I^2^ = 16%
>1,000	5	1.25	1.02, 1.52	*P* = 0.03	*P* = 0.05; I^2^ = 58%
**Adjustment of BMI**					
Adjusted	5	1.40	1.23, 1.60	*P* < 0.001	*P* = 0.15; I^2^ = 41%
Unadjusted	3	1.36	0.89, 2.08	*P* = 0.16	*P* = 0.02; I^2^ = 74%
**Adjustment of physical activity**					
Adjusted	5	1.48	1.29, 1.71	*P* < 0.001	*P* = 0.53; I^2^ = 0%
Unadjusted	3	1.14	0.81, 1.61	*P* = 0.46	*P* = 0.02; I^2^ = 75%

### Unprocessed Red Meat Consumption and the Risk of MetS

**Figure 3:**
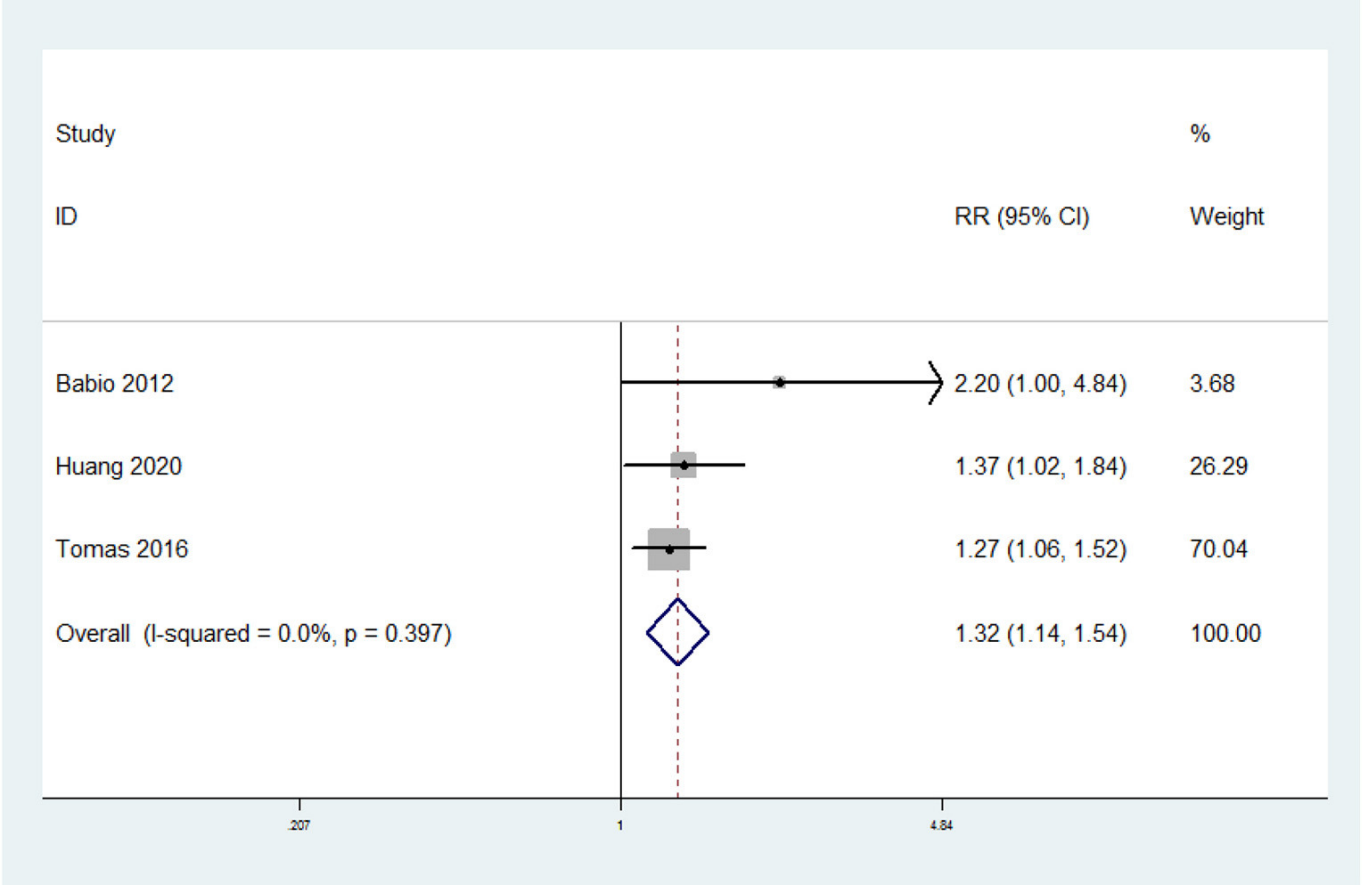
Forest plot of meta-analysis: overall multi-variable adjusted RR of MetS for the highest vs. lowest category of unprocessed red meat consumption.

Three prospective cohort studies were included. The overall multi-variable adjusted RR showed that unprocessed red meat consumption was associated with a higher risk of MetS (RR = 1.32 95% CI: 1.14–1.54; *P* = 0.0003) (Figure 3). We did not find a substantial level of heterogeneity among the studies (*P* = 0.397, I^2^ = 0%). No publication bias was observed according to the Begg's rank-correlation test (*P* = 0.296) and the Egger's test (*P* = 0.07).

### Processed Red Meat Consumption and the Risk of MetS

**Figure 4:**
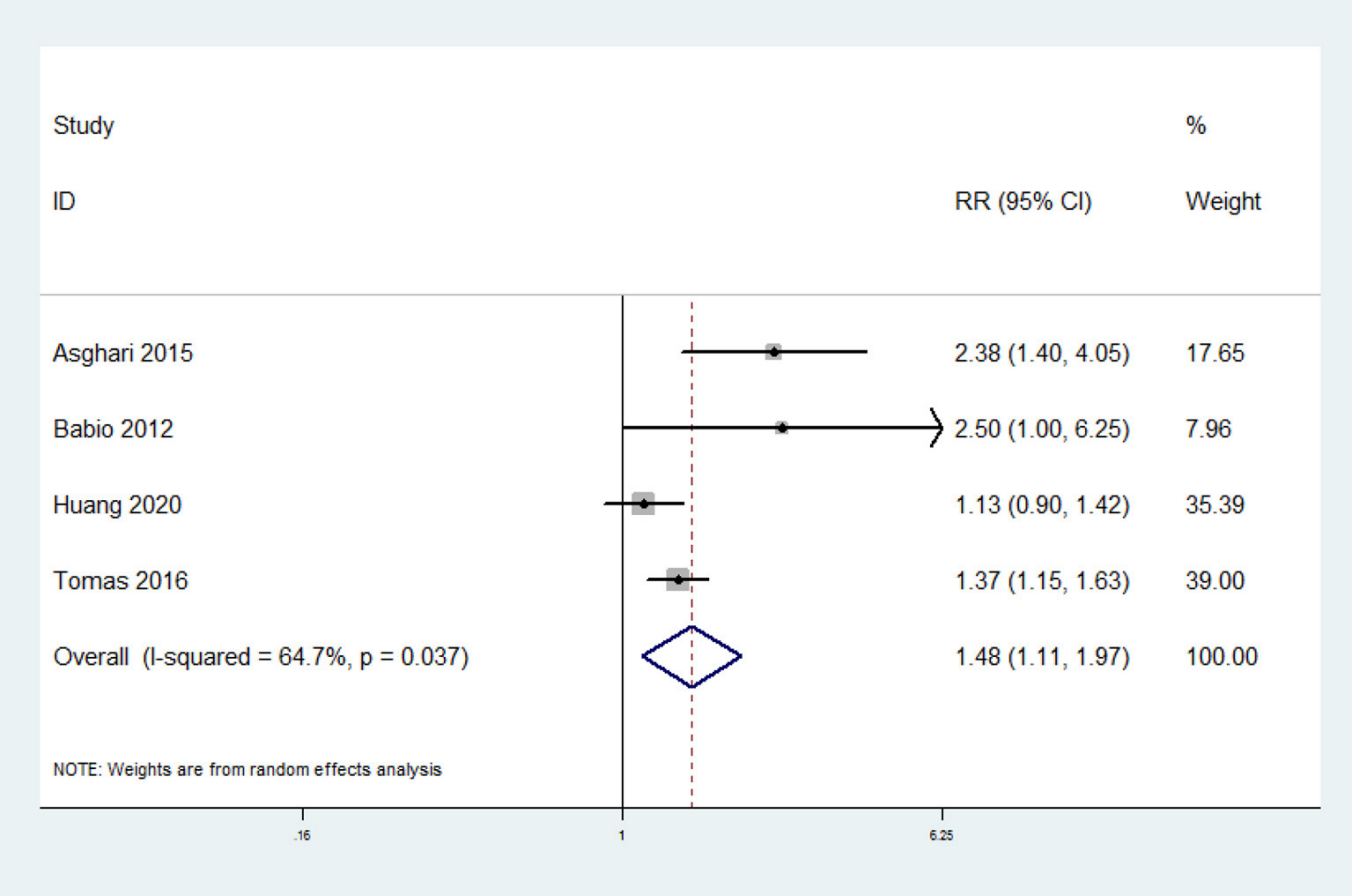
Forest plot of meta-analysis: overall multi-variable adjusted RR of MetS for the highest vs. lowest category of processed red meat consumption.

Four prospective cohort studies were included. The overall multi-variable adjusted RR showed that processed red meat consumption was associated with a higher risk of MetS (RR = 1.48, 95% CI: 1.11–1.97; *P* = 0.007) (Figure 4). We found a substantial level of heterogeneity among the studies (*P* = 0.037, I^2^ = 64.7%). No publication bias was observed according to the Begg's rank-correlation test (*P* = 0.734) and the Egger's test (*P* = 0.259).

### Poultry Consumption and the Risk of MetS

**Figure 5:**
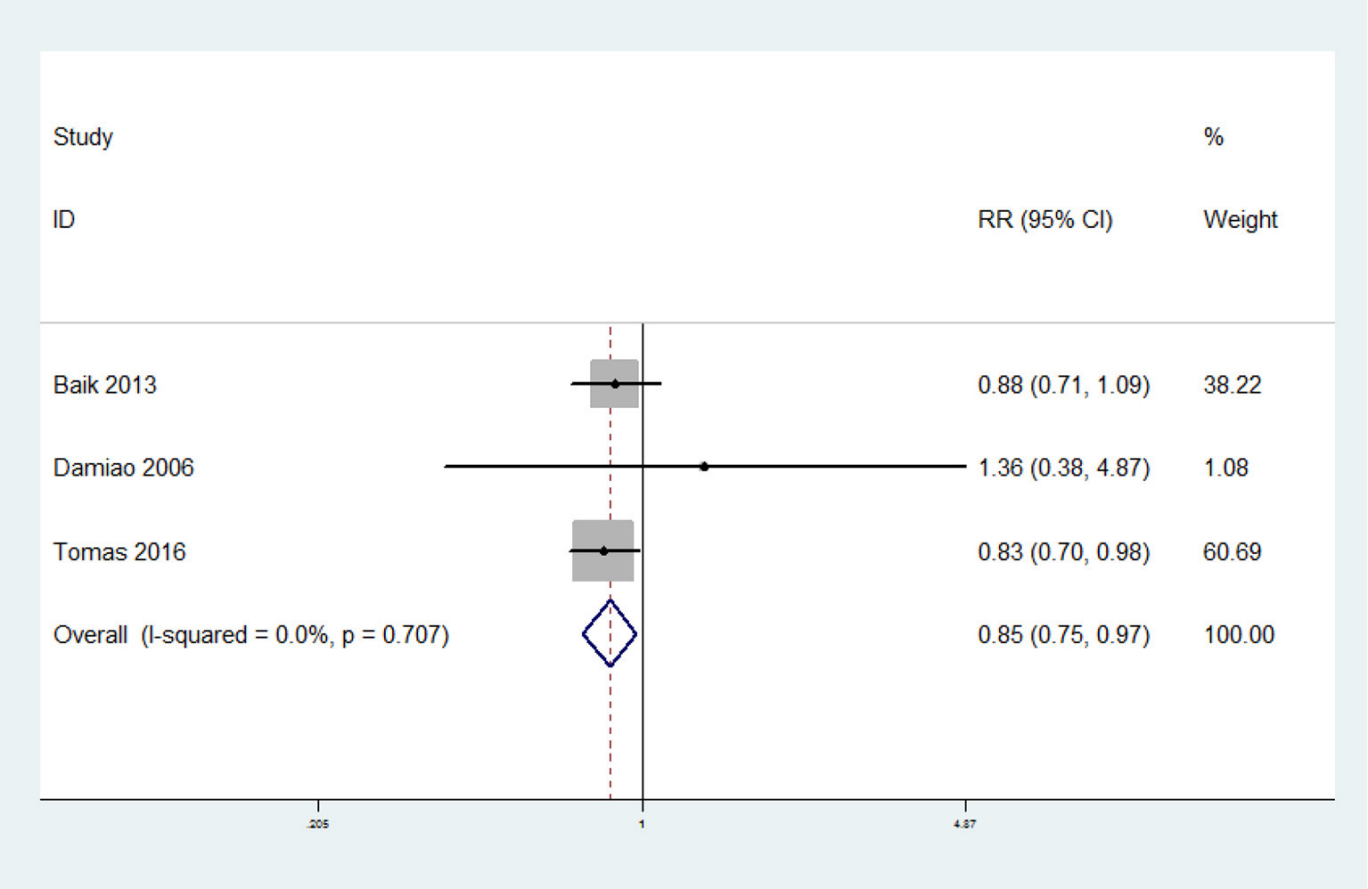
Forest plot of meta-analysis: overall multi-variable adjusted RR of MetS for the highest vs. lowest category of poultry consumption.

Three prospective cohort studies were included. The overall multi-variable adjusted RR showed that poultry consumption was associated with lower risk of MetS (RR = 0.85, 95% CI:.75–0.97; *P* = 0.02) (Figure 5). We did not find a substantial level of heterogeneity among the studies (*P* = 0.707, I^2^ = 0%). No publication bias was observed according to the Begg's rank-correlation test (*P* = 0.296) and the Egger's test (*P* = 0.215).

## Discussion

In this meta-analysis, a total of nine prospective cohort studies were identified (Table 3). The results showed that red meat (processed and unprocessed) consumption was associated a higher risk of MetS, whereas, poultry consumption was associated with a lower risk of MetS.

**Table 3:** Summarized RR of MetS for highest vs. lowest category of exposure.

**Study**	**Pooled RR**	**95% CI**
**Red meat**		
Damiao 2006	3.18	0.87, 11.62
Babio 2012	2.70	1.10, 6.63
Baik 2013	1.01	0.79, 1.29
Shang 2016	1.47	1.01, 2.14
Tomas 2016	1.46	1.22, 1.75
Esfandiar 2019	0.87	0.56, 1.35
Huang 2020	1.41	1.05, 1.89
Yuzbashian 2021	1.55	1.13, 1.62
**Unprocessed red meat**		
Babio 2012	2.20	1.00, 4.84
Tomas 2016	1.27	1.06, 1.52
Huang 2020	1.37	1.02, 1.84
**Processed red meat**		
Babio 2012	2.50	1.00, 6.25
Asghari 2015	2.38	1.40, 4.05
Tomas 2016	1.37	1.15, 1.63
Huang 2020	1.13	0.90, 1.42
**Poultry**		
Baik 2013	0.88	0.71, 1.09
Tomas 2016	0.83	0.70, 0.98
Damiao 2006	1.36	0.38, 4.87

The mechanism on how red meat consumption contributes to the development of MetS may be explained as follows. First, as a major compound in red meat, saturated fatty acids (SFAs) are associated with higher body weight in animals (28), which suggests that SFAs may contribute to the etiology of metabolic disorders. Second, the heme iron in red meat is also related to MetS (29). Iron can potentially induce oxidative stress and in turn, lead to insulin resistance (30). Third, some additives in the processed red meat may also contribute to MetS. Nitrites and nitrates can be converted into nitrosamines, which may increase diabetes risk in animal models (31). Moreover, blood nitrites are found to play important roles in impaired insulin response and endothelial dysfunction in adults (32). Lastly, the sodium from processed red meat may contribute to the risk of hypertension (33).

As mentioned in the introduction, the components in red meat and poultry, and their procession are rather different. The biological effect of meat may, therefore, vary greatly. Indeed, the relationship between meat consumption (red meat and poultry were considered as a whole) and MetS has been investigated by several cross-sectional studies with conflicting results (34–38). This discrepancy might be attributed to the synthetic effect of red meat and poultry. The results showed a direct opposite effect of red meat vs. poultry consumption on the risk of MetS. Furthermore, a conference abstract suggested that higher fresh fatty red meat (but not lean red meat) consumption was negatively associated with MetS (39). Therefore, a detailed specification of the meat component is needed in further study.

It should be noted that the classification of exposure varied greatly among the studies, which may influence the results of this study. For example, the definition of estimates varies according to the different definitions of exposure (inconsistent metric). However, it is not sensible to investigate the inconsistent estimates. It could be partly addressed by randomized controlled trials (their general consistency of exposure is often stronger than that in observational study). However, unfortunately, no relevant randomized controlled trial has been performed yet. As a consequence, further, well-designed randomized controlled trials are still needed.

Of note, one cross-sectional study has tried to conduct a meta-analysis after reporting their original data (16). However, it failed to demonstrate the association between red meat consumption and the risk of MetS (only three prospective cohort studies were included). Moreover, the risk of MetS and unprocessed red meat was not considered either. This study was, therefore, employed to address the issues above. The results showed that both processed and unprocessed red meat consumption was associated with a higher risk of MetS, whereas, poultry consumption was associated with a lower risk of MetS. Interestingly, the positive relationship between red meat consumption and the risk of MetS was only obtained in >5 years follow-up, non-NCEP ATP III, Non-Asia, adjustment of BMI, and physical activity studies. However, the pooled RR showed a relative similar strength of association, and the influence of significant heterogeneity could not be fully addressed. Thus, further studies are still needed to elucidate the effect of follow-up, diagnostic criteria of MetS, geographical region, BMI, and physical activity on the relationship between meat consumption and the risk of MetS.

This study has several strengths: First, this is the first meta-analysis of prospective cohort studies on the association of red meat (both processed and unprocessed) and poultry consumption with the risk of MetS, which could reflect a causal relationship. Second, the included studies are analyzed based on the adjusted results and large samples. On the other hand, we should also acknowledge the limitations of this study. First, the substantial level of heterogeneity may distort the reliability of the results. Second, only a small number of prospective cohort studies are identified because of limited relevant evidence. Third, the classification of exposure varies greatly among individuals. Fourth, the selection of adjusted factors and definition of MetS were not uniform. Fifth, one included study reported the combined data for poultry and rabbit (22). Last but not the least, no study has specified the fatty or lean red meat, so some issues cannot be addressed. Taken together, this study may be restricted by these limitations.

## Conclusion

The current evidence indicates that red meat (processed and unprocessed) consumption is associated with higher risk of MetS, whereas, poultry consumption is associated with lower risk of MetS. More well-designed randomized controlled trials are still needed to address the issues further.

## Data Availability Statement

The raw data supporting the conclusions of this article will be made available by the authors, without undue reservation.

## Author Contributions

YZ conceived the idea, performed the statistical analysis, and drafted this meta-analysis. YZ and JD selected and retrieved relevant papers. YZ and JL assessed each study. HG was the guarantor of the overall content. All authors revised and approved the final manuscript.

## Conflict of Interest

The authors declare that the research was conducted in the absence of any commercial or financial relationships that could be construed as a potential conflict of interest.
